# A Comprehensive Analysis of HAVCR1 as a Prognostic and Diagnostic Marker for Pan-Cancer

**DOI:** 10.3389/fgene.2022.904114

**Published:** 2022-06-08

**Authors:** Sheng Liu, Wenting Tang, Jing Cao, Mei Shang, Hengchang Sun, Jiao Gong, Bo Hu

**Affiliations:** ^1^ Department of Laboratory Medicine, Third Affiliated Hospital of Sun Yat-sen University, Guangzhou, China; ^2^ State Key Laboratory of Oncology in South China, Collaborative Innovation Center for Cancer Medicine, Sun Yat-sen Cancer Center, Guangzhou, China; ^3^ Department of Molecular Diagnostics, Sun Yat-sen Cancer Center, Guangzhou, China; ^4^ Department of Infectious Diseases, Third Affiliated Hospital of Sun Yat-sen University, Guangzhou, China

**Keywords:** havcr1, prognosis, diagnostic, liver hepatocellular carcinoma, pancreatic adenocarcinoma

## Abstract

Hepatitis A virus cellular receptor (HAVCR1) is a type-1 integral membrane glycoprotein that plays a key role in immunity and renal regeneration and is abnormally expressed in various tumor types. Nonetheless, the function of HAVCR1 in pan-cancer remains unknown. In this study, we comprehensively analyzed the expression and promoter methylation level of HAVCR1 and assessed the immune cell infiltration, correlation between stromal and immune cell admixture, CD (Cluster of Differentiation) and HAVCR1 expression and prognostic value of HAVCR1 mRNA expression in Liver hepatocellular carcinoma (LIHC) and Pancreatic adenocarcinoma (PAAD). Our results showed that HAVCR1 was overexpressed while the promoter methylation of HAVCR1 was decreased in Liver hepatocellular carcinoma and Pancreatic adenocarcinoma. HAVCR1 was associated with increased infiltration of B cells, CD8 cells, macrophages, neutrophils and Dendritic cells in Liver hepatocellular carcinoma and Pancreatic adenocarcinoma. HAVCR1 expression was positively correlated with the immune, stromal and estimate scores of Pancreatic adenocarcinoma and the stromal and estimate scores of Liver hepatocellular carcinoma. Furthermore, HAVCR1 expression was correlated with other immune molecules such as HHLA2 (Human endogenous retrovirus-H long terminal repeat-associating protein 2), CD44 and TNFRSF4 (TNF Receptor Superfamily Member 4) in Liver hepatocellular carcinoma and Pancreatic adenocarcinoma. During Kaplan-Meier analysis, high HAVCR1 expression in Liver hepatocellular carcinoma and Pancreatic adenocarcinoma correlated with poor survival. A marginally significant *p*-value (*p* = 0.051) was obtained when the relationship between HAVCR1 expression in Liver hepatocellular carcinoma and prognosis was analyzed, attributed to the small sample size. Overall, we provided compelling evidence that HAVCR1 could be a prognostic and diagnostic marker for Liver hepatocellular carcinoma and Pancreatic adenocarcinoma.

## Introduction

The Hepatitis A virus cellular receptor (HAVCR1), also known as T-cell immunoglobulin mucin receptor 1 (TIM-1) and kidney injury molecule 1 (KIM-1), has been established to play an important role in intercellular interactions, such as recognition, immune activation, tight junction, even cancer biology (Telford et al., 2017; Ginès et al., 2018; Evans and Liu, 2020). HAVCR1 has an N-terminal Cys-rich immunoglobulin variable- (IgV) like domain, a mucin-like domain, a transmembrane domain, and a tyrosine phosphorylation motif intracellular tail used for signal transduction (Tietjen et al., 2017). Although different ligands can bind to HAVCR1, it is most predominantly bound by TIM4 and phosphatidylserine. However, P-selectin and S-selectin have also been reported to be potential ligands (Du et al., 2016).

HAVCR1 is a costimulatory molecule expressed on the surface of immune cells, which promotes activation and proliferation of immune cells, cytokine secretion and regulates the immune response (Guo et al., 2019; Evans and Liu, 2020; Xiao et al., 2020). In this regard, HAVCR1 can alter the function of CD8^+^ T cells and NK cells for an effective antitumor immune response (Lee et al., 2010; Forbes et al., 2021). An increasing body of evidence suggests that the positive and negative regulation of HAVCR1 by ligand-binding is essential for maintaining immune homeostasis (Xiao et al., 2007; Brooks et al., 2015; Du et al., 2016).

Tumor immunity involves a cell-mediated (type 1) immune response by Th1 cells, cytotoxic T lymphocytes, NK cells and NKT cells. Notably, CD8^+^ T cells are the main effector cells in tumor immunity (Liu et al., 2021a; Liu et al., 2021b; Song et al., 2021). In most cases, tumor infiltration by CD8^+^ T cells is a favorable prognostic indicator. However, during immune inhibition, CD8^+^ T cells can indirectly help tumors escape immunity by increasing the expression of Treg cells and myeloid-derived suppressor cells (MDSCs) (Kurebayashi et al., 2021; Tengesdal et al., 2021). Besides, low expression of co-stimulation molecules of type 1 lymphocytes can induce immune escape or immune tolerance (Labiano et al., 2016; Panneton et al., 2019). The co-stimulation signal is reportedly vital for the activation and performance of CD8^+^ T cells (Weulersse et al., 2020). Binding to the transmembrane protein HAVCR1 can activate the IL-6/STAT3 signaling pathway to regulate cell growth, proliferation, and metabolism. Hence, the IL-6/STAT3 signaling pathway is a potential tumor therapeutic target (Cuadros et al., 2014). Taken together, HAVCR1 acts as a costimulatory molecule during tumor treatment that can enhance the antitumor effect of lymphocytes and induce tumor microenvironmental changes for effective antitumor immunity.

The aberrant expression of HAVCR1 (also known as KIM1) was first documented in kidney injury, then in autoimmune and allergic diseases (Meyers et al., 2005). Over the years, although unprecedented scientific progress has been achieved, it has only been established that HAVCR1 plays a key role in the immune response as a costimulatory molecule. Little is currently known about the function of HAVCR1 in immune cells, such as B cells, T cells, NK cells, DC cells, and Macrophages. Herein, we sought to explore the function of HAVCR1 in tumors, especially in Liver hepatocellular carcinoma and Pancreatic adenocarcinoma.

## Material and Methods

### Expression of HAVCR1 in Different Tumors

The Oncomine database is widely acknowledged to have powerful analytical capabilities for calculating gene expression characteristics, clusters, and genomic modules (https://www.oncomine.org/resource/login.html). In the present study, Oncomine was used to assess the expression of HAVCR1 in various tumors. Correlation analysis between HAVCR1 expression in tumor and normal tissue was stratified by tumor stages and promoter methylation level of HAVCR1 in Liver hepatocellular carcinoma and Pancreatic adenocarcinoma using UALCAN (http://ualcan.path.uab.edu/)[25]. UALCAN is an interactive website used to perform in-depth analyses of cancer OMICS data, especially TCGA gene expression data and analyze the relative expression of a query gene(s) across tumor and normal samples, as well as in various tumor subgroups, based on individual cancer stages, tumor grade or other clinicopathological features. The statistical significance of each comparison performed is provided in the figures.

### The Relationship Between Overall Survival and HAVCR1 Expression in Tumors

The correlation between HAVCR1 expression and survival in Liver hepatocellular carcinoma and Pancreatic adenocarcinoma was analyzed by the Kaplan-Meier plotter (http://kmplot.com/analysis/index.php?p=service&cancer=pancancer_rnaseq). The log-rank *p*-value and a hazard ratio (HR) with a 95% confidence interval were also calculated.

### Immune Microenvironment Assessment

The Tumor Immune Estimation Resource (TIMER) database (https://cistrome.shinyapps.io/timer/) contains 32 cancers from the Cancer Genome Atlas (TCGA) with a total of 10,897 samples. The immune cells in the TIMER database consist of CD4+T cells, CD8+T cells, B cells, neutrophils, macrophages, and dendritic cells. The TIMER module sCNA was used to evaluate the abundance of infiltrated immune cells in Liver hepatocellular carcinoma and Pancreatic adenocarcinoma. sCNA module enables the comparison of tumor infiltration levels among tumors with different somatic copy number alterations for a given gene. The infiltration level for each SCNA category is compared with the normal level using a two-sided Wilcoxon rank-sum test. In addition, the R package “ESTIMATE” was used to estimate stromal and immune cells in Liver hepatocellular carcinoma and Pancreatic adenocarcinoma tissues by calculating the stromal score, immune score and ESTIMATE score of each sample.

### The Relationship Between HAVCR1 and Other Immune Checkpoints

The R package “corrplot” provides a visual exploratory tool on correlation matrix that supports automatic variable reordering to help detect hidden patterns among variables. In the present study, “corrplot” was used to explore the relationship between HAVCR1 and other key molecules. For example, immune checkpoint molecules and HAVCR1 may have a synergistic effect.

### Diagnostic Prediction

IBM SPSS Statistics 25 was used to plot the ROC curves based on the expression of HAVCR1 in tumor tissues and normal tissues. The ROC curves were used to evaluate the predictive power of HAVCR1 for the survival of Liver hepatocellular carcinoma and Pancreatic adenocarcinoma patients. An area under the ROC curve (AUC) value >0.7 had diagnostic value.

### Immunohistochemistry

The normal tissues and Liver hepatocellular carcinoma tissues were fixed in formalin and embedded with paraffin and then cut into 4 µm-thick sections and placed on polylysine-coated slides. The slides were heated at 50°C for 3 h, deparaffinized in xylene, rehydrated through graded ethanol, quenched for endogenous peroxidase activity in 0.3% hydrogen peroxide, and processed for antigen retrieval by microwave heating in 10 mM citrate buffer (pH6.0). The slides were incubated with the polyclonal rabbit Ab against HAVCR1 overnight at 4°C; the primary antibody was diluted at 1:300 in Antibody Diluent with Background Reducing Components (DakoCytomation, Glostrup, Denmark). Immunostaining for HAVCR1 was performed using ChemMate DAKO EnVision Detection Kit, Peroxidase/DAB, Rabbit/Mouse (code K 5007, DakoCytomation), which yielded a brown colored precipitate around the antigen. Then, the slides were counterstained with hematoxylin (Zymed Laboratories, South San Francisco, CA, United States) and mounted in a non-aqueous mounting medium. No primary antibody controls were set up for all IHC staining experiments.

All slides were independently evaluated by two experienced pathologists. HAVCR1 protein expression grading was conducted by analyzing the intensity and percentage of positive cells in five different areas. The staining intensity was scored using a 4-point scale: 0 for no staining, 1 for mild staining, 2 for moderate staining, and 3 for intense staining. The percentage of positive cells was scored with a 4-point scale: 0 for no staining of cells in any microscopic areas, 1 for 1–30%, 2 for 30–60%, and 3 for over 60%. The total score of HAVCR1 staining intensity was calculated by multiplying the intensity score by the percentage score. The relevant characteristics of the study subjects are shown in Supplementary Table S1.

## Results

### HAVCR1 Expression Is Dysregulated in Tumors

**FIGURE 1:**
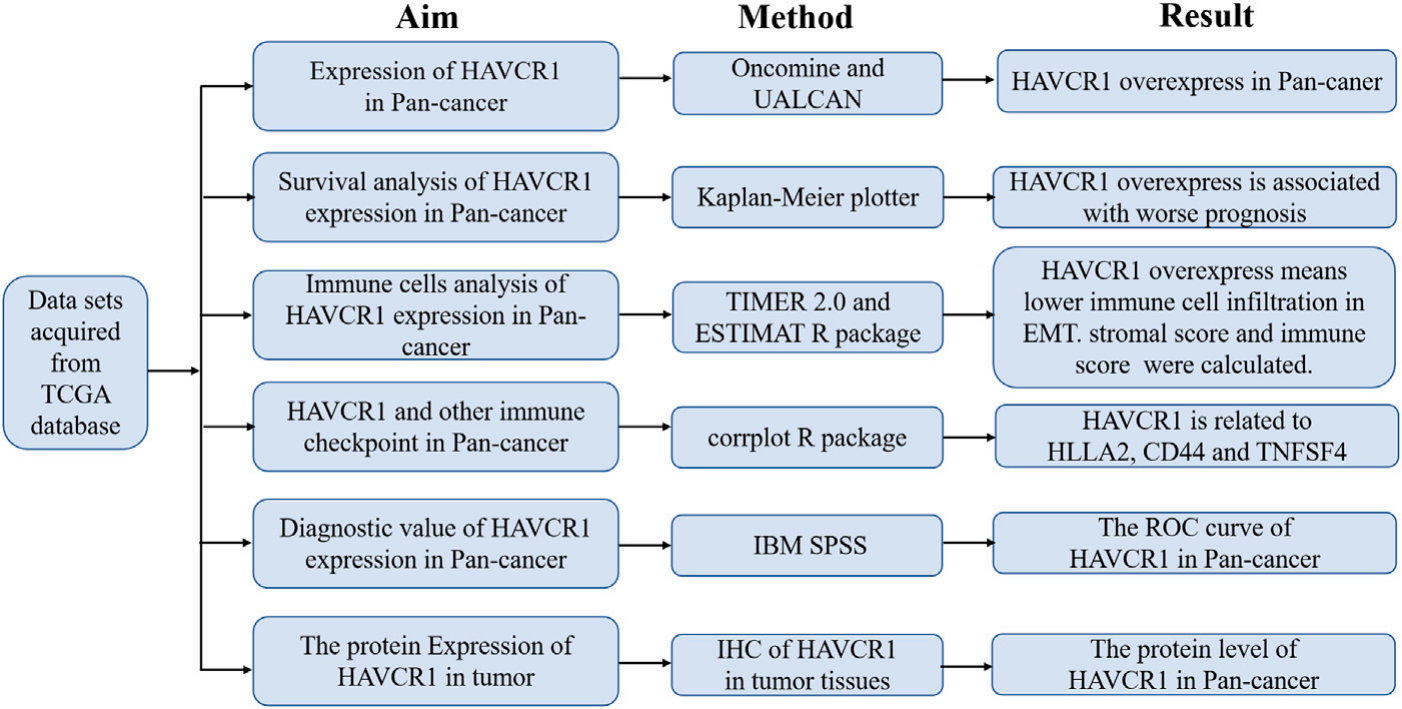
Workflow chart for this study.

**FIGURE 2:**
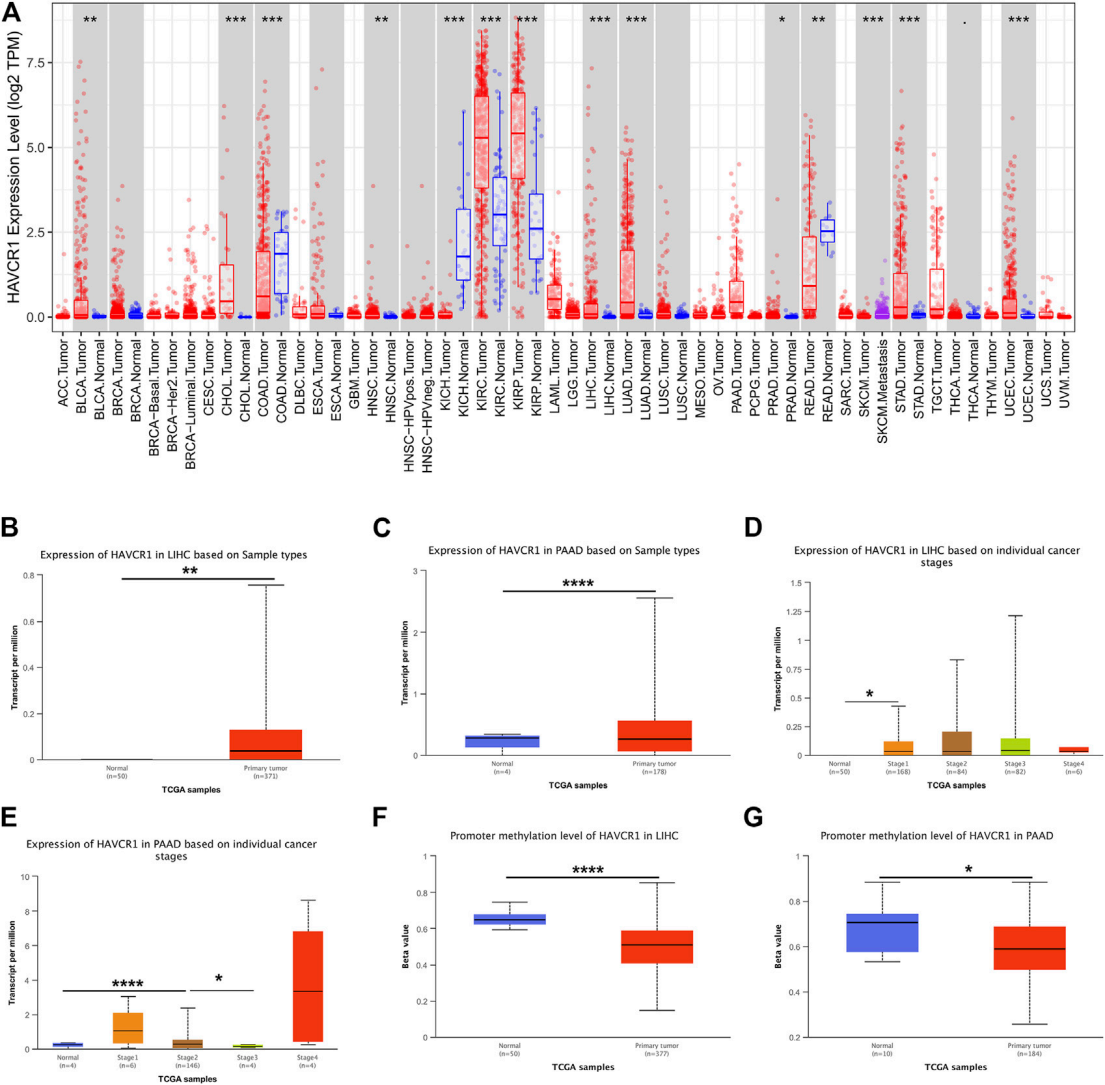
HAVCR1 was overexpressed in tumor tissues compared to normal tissue in Pan-cancer. **(A)** HAVCR1 was aberrantly expressed in numerous tumors and was upregulated in most tumors. **(B,C)** HAVCR1 mRNA expression in tumor and normal tissue in Liver hepatocellular carcinoma and Pancreatic adenocarcinoma. **(D,E)** HAVCR1 mRNA expression in tumor and normal tissue in different stages of Liver hepatocellular carcinoma and Pancreatic adenocarcinoma. **(F,G)** Promoter methylation levels of HAVCR1 were mostly decreased in Liver hepatocellular carcinoma and Pancreatic adenocarcinoma. ^∗^ *p* < 0.05, ^∗∗^ *p* < 0.01, ^∗∗∗^ *p* < 0.001, ****p < 0.001.

HAVCR1 has been established as a sensitive early marker for kidney injury. However, we aimed to uncover its function in tumors in the present study. Our study flowchart is illustrated in Figure 1. HAVCR1 expression in TCGA pan-cancer was analyzed using TIMER (https://cistrome.shinyapps.io/timer/). HAVCR1 expression was dysregulated in numerous tumor types: BLCA (Bladder Urothelial Carcinoma, *p* < 0.01), CHOL (Cholangiocarcinoma, *p* < 0.001), COAD (Colon adenocarcinoma, *p* < 0.001), HNSC (Head and Neck squamous cell carcinoma, *p* < 0.01), KICH (Kidney Chromophobe, *p* < 0.001), KIRC (Kidney renal clear cell carcinoma, *p* < 0.001), KIRP (Kidney renal papillary cell carcinoma, *p* < 0.001), LIHC (Liver hepatocellular carcinoma, *p* < 0.001), LUAD (Lung adenocarcinoma, *p* < 0.001), PRAD (Prostate adenocarcinoma, *p* < 0.05), READ (Rectum adenocarcinoma, *p* < 0.01), SKCM (Skin Cutaneous Melanoma, *p* < 0.001), STAD (Stomach adenocarcinoma, *p* < 0.001), UCEC (Uterine Corpus Endometrial Carcinoma, *p* < 0.001)) (Figure 2A). Given the poor prognostic role of HAVCR1 in various tumor types, we focused on the role of HAVCR1 in Liver hepatocellular carcinoma (*p* < 0.01) and Pancreatic adenocarcinoma (*p* < 0.001). The overexpression of HAVCR1 was then confirmed by UALCAN (Figures 2B,C) (Chandrashekar et al., 2017). Subsequently, we analyzed HAVCR1 expression in different stages of tumors. HAVCR1 was upregulated in stage 1 compared to normal tissue in Liver hepatocellular carcinoma patients (*p* < 0.05, Figure 2D). Moreover, HAVCR1 was upregulated in stage 2 (*p* < 0.001) and stage 3 (*p* < 0.05, Figure 2E) compared to normal tissue in Pancreatic adenocarcinoma patients.

DNA demethylation and gene-specific promoter DNA methylation have been documented across cancer types. Studies on DNA methylation changes have led to a broader understanding of tumorigenesis and demonstrated their role as promising candidates for early tumor detection or high-risk subject screening. In this study, we assessed the role of the promoter methylation level of HAVCR1 as a biomarker for early tumor detection and their potential clinical applications in Liver hepatocellular carcinoma and Pancreatic adenocarcinoma. We found that the promoter methylation level of HAVCR1 was significantly downregulated in Liver hepatocellular carcinoma and Pancreatic adenocarcinoma (Figures 2F,G), which might lead to HAVCR1 overexpression.

### Prognostic Value of HAVCR1 mRNA Expression in Liver Hepatocellular Carcinoma and Pancreatic Adenocarcinoma

**FIGURE 3:**
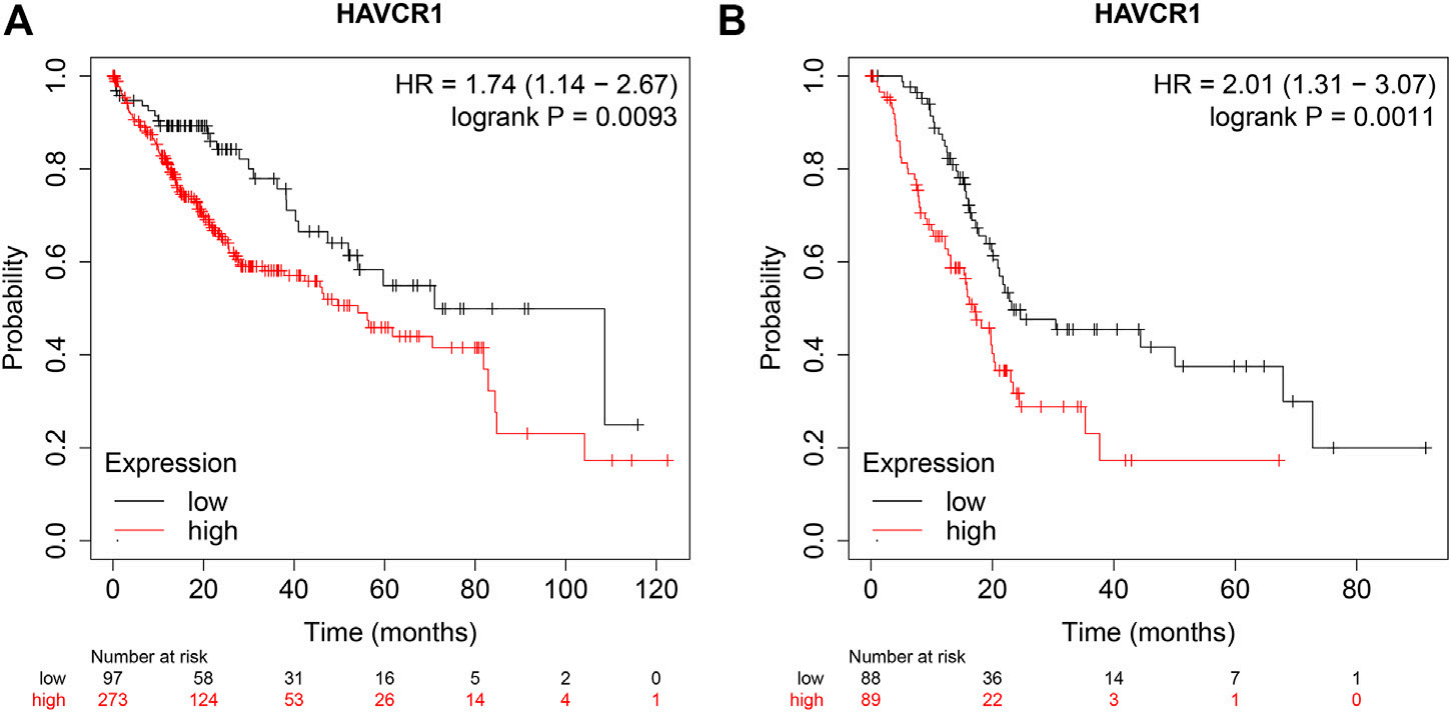
The OS curve of HAVCR1 expression in Liver hepatocellular carcinoma and Pancreatic adenocarcinoma. **(A,B)** Database Analysis The correlation between HAVCR1 expression and survival in pancreatic cancer was analyzed by Kaplan-Meier plotter; HAVCR1 overexpression was associated with a poor prognosis in Liver hepatocellular carcinoma and Pancreatic adenocarcinoma.

Furthermore, we analyzed the prognostic value of HAVCR1 in gastrointestinal tumors using KM-Plot (http://kmplot.com/analysis/). High expression of HAVCR1 correlated with a worse prognosis in Liver hepatocellular carcinoma (HR = 1.74, 95% CI: 1.14–2.67, and *p* = 0.0093, Figure 3A) and Pancreatic adenocarcinoma (HR = 2.01, 95% CI: 1.31–3.07, and *p* = 0.0011, Figure 3B). These results suggest that HAVCR1 may serve as useful biomarkers for predicting Liver hepatocellular carcinoma and Pancreatic adenocarcinoma prognosis.

### High Expression of HAVCR1 Correlated With Immune Cell Infiltration in Liver Hepatocellular Carcinoma and Pancreatic Adenocarcinoma

**FIGURE 4:**
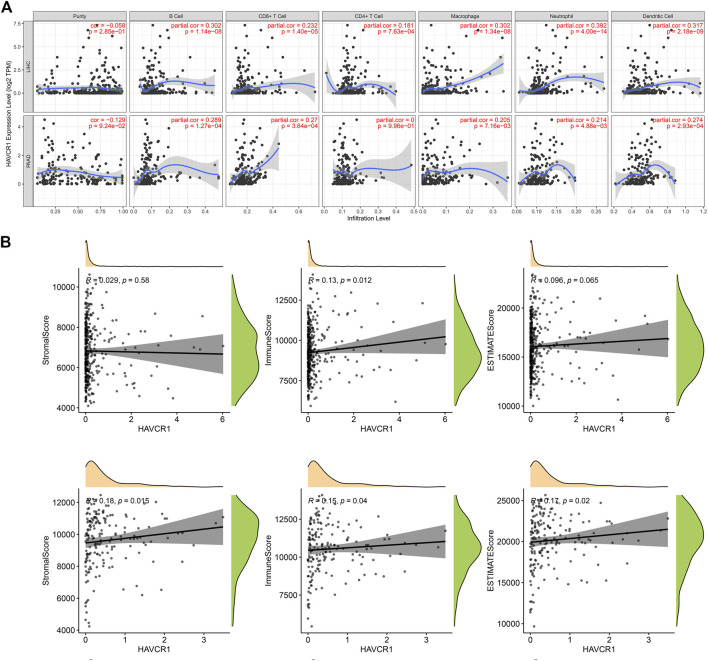
Analysis of the relationship between immune cells infiltration and HAVCR1 expression in Liver hepatocellular carcinoma and Pancreatic adenocarcinoma. **(A)** TIMER2.0 was used to analyze the expression level of HAVCR1 in immune cells, including B cell, CD8^+^ T cell, CD4^+^ T cell, macrophage, neutrophil cell and dendritic cell. High HAVCR1 expression correlated with immune cell infiltration in Liver hepatocellular carcinoma and Pancreatic adenocarcinoma. **(B)** Correlation of scores in patients with HAVCR1 in Liver hepatocellular carcinoma and Pancreatic adenocarcinoma, stromal and immune cell admixture from expression data correlated to HAVCR1 expression. *p* values <0.05 were statistically significant.

HAVCR1 is reportedly important in tumorigenesis for its role in regulating the function of immune cells. TIMER2.0 was used to analyze the correlation between HAVCR1 gene expression and immune cell composition. In Liver hepatocellular carcinoma and Pancreatic adenocarcinoma, high HAVCR1 expression correlated with immune cell infiltration (Figure 4A). It is widely acknowledged that the tumor microenvironment tissue is a mixture of tumor-infiltrating immune cells, stromal cells, and endothelial cells. Stromal cells and immune cells play an important role in the progression and treatment of tumors (Gajewski et al., 2013; Shi et al., 2017). In our study, we estimated the immune score, stromal score and estimate score using the R package “ESTIMATE” and further calculated their correlation with HAVCR1. We found that overexpression of HAVCR1 correlated with the stromal score, immune score and estimate score in Liver hepatocellular carcinoma and Pancreatic adenocarcinoma (Figure 4B).

### The Correlation Between HAVCR1 and Immune Molecules in Liver Hepatocellular Carcinoma and Pancreatic Adenocarcinoma

**FIGURE 5:**
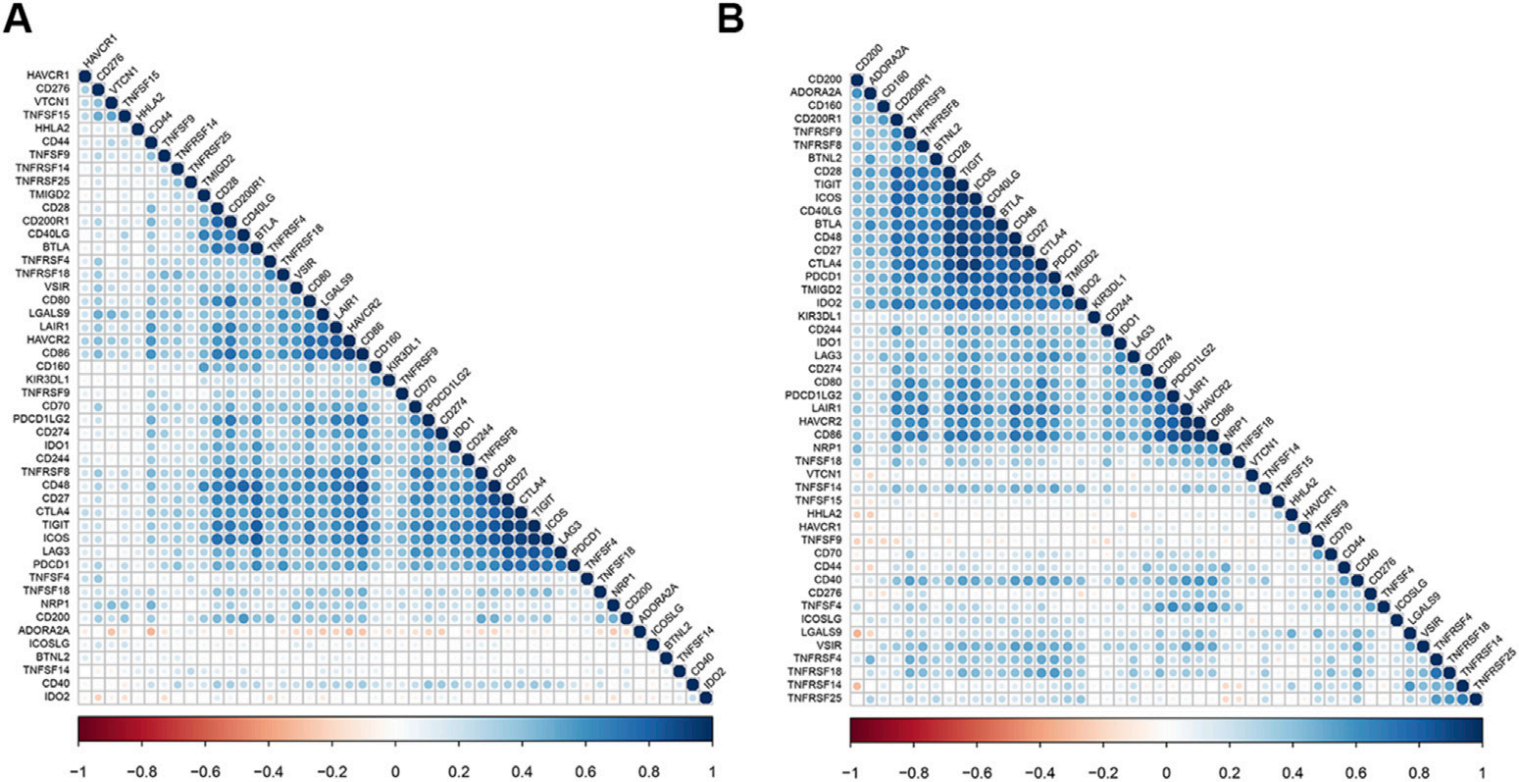
HAVCR1 is correlated with other checkpoints in Liver hepatocellular carcinoma and Pancreatic adenocarcinoma. Correlation between HAVCR1 and marker gene sets of immune cells across cancers in TCGA. Our results showed that HHLA2, CD44 and TNFSF4 were correlated with HAVCR1 expression in Liver hepatocellular carcinoma **(A)** and Pancreatic adenocarcinoma **(B)**.

We calculated the correlation between HAVCR1 and immune checkpoint molecules, known to be important for tumorigenesis or tumor treatment, and visualized them by R package “corrplot”. We found that HAVCR1 was associated with HHLA2, CD44 and TNFRSF4 in Liver hepatocellular carcinoma and Pancreatic adenocarcinoma (Figures 5A,B).

### Diagnostic Value of HAVCR1 in Liver Hepatocellular Carcinoma and Pancreatic Adenocarcinoma

**FIGURE 6:**
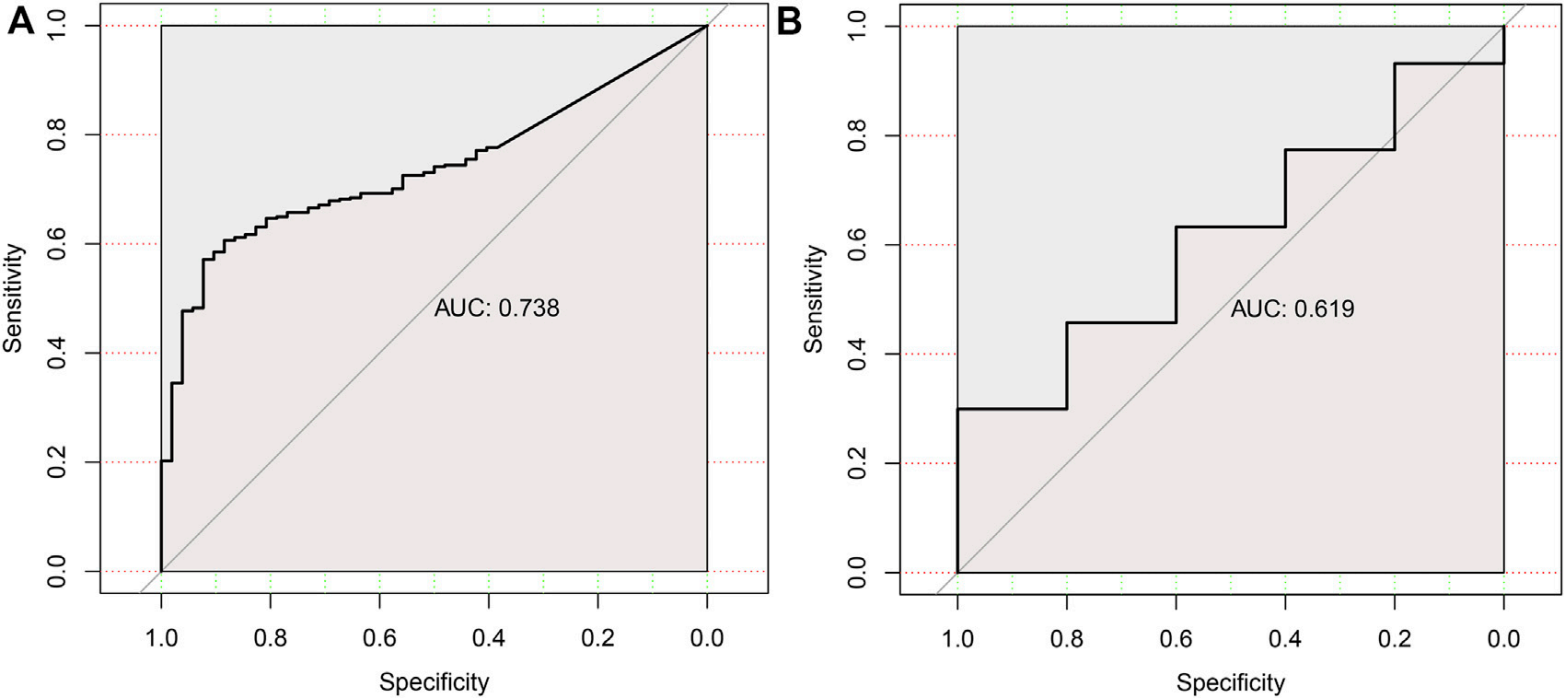
The ROC curve of HAVCR1 expression in Liver hepatocellular carcinoma and Pancreatic adenocarcinoma.The ROC curves were used to evaluate the predictive power of the HAVCR1 of 5-years survival in Liver hepatocellular carcinoma and Pancreatic adenocarcinoma. The AUC of the ROC curve was 0.738 in Liver hepatocellular carcinoma **(A)** and 0.619 in Pancreatic adenocarcinoma **(B)**.

To assess the diagnostic performance of HAVCR1 in tumors, receiver operating characteristic curve analysis was conducted. The ROC curves revealed that the AUCs of HAVCR1 for predicting the 5-years survival of Liver hepatocellular carcinoma and Pancreatic adenocarcinoma patients were 0.738 and 0.619, respectively (Figures 6A,B).

### Prognostic Value of the Protein Expression of HAVCR1 in Liver Hepatocellular Carcinoma by IHC

**FIGURE 7:**
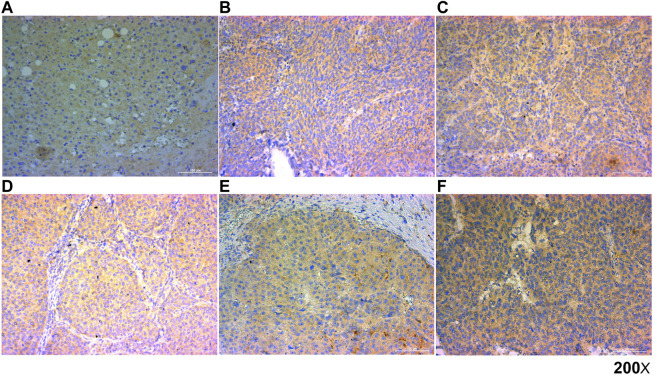
Representative images of HAVCR1 expression in Liver hepatocellular carcinoma by IHC. Representative images of HAVCR1 expression in normal tissue **(A)** and Liver hepatocellular carcinoma tissues **(B–F)** captured by a Nikon microscope. The scale bar is 100 μm.

Finally, we analyzed the prognostic value of HAVCR1 in Liver hepatocellular carcinoma by IHC staining. We analyzed the correlation between the expression of HAVCR1 in normal tissues (n = 3), Liver hepatocellular carcinoma tissues (*n* = 46) and prognosis. The clinical information of HCC patients is provided in Supplementary Table S1. Our results showed that HAVCR1 was upregulated in Liver hepatocellular carcinoma (Figures 7B–F) compared to normal tissues (Figure 7A), and a marginally significant *p*-value (*p* = 0.051) was obtained during survival analysis which can be attributed to the small sample size. The correlation between clinical characteristics and HAVCR1 expression is shown in Supplementary Table S1. Importantly, HCC patients with high expression of HAVCR1 exhibited high recurrence and mortality rates (Supplementary Table S1).

## Discussion

There is ample evidence suggesting that HAVCR1 expression is dysregulated across tumors. However, its function in tumor development warrants further investigation. It has been shown that HAVCR1 is involved in the formation, maintenance and function of tight junctions, key components of tumor metastasis (Telford et al., 2017). Moreover, HAVCR1 expression has been documented in immune cells and immune response activation and regulation (Naeini et al., 2020). In addition, given that the HAVCR1 ectodomain is cleaved and released during urine formation, it is a valuable biomarker for kidney injury (Tanase et al., 2019). Furthermore, the HAVCR1 ectodomain can activate the IL-6/STAT3 pathway to induce HIF-1α-mediated angiogenesis, and its Ig-like domain can bind to various cells involved in metastasis. HAVCR1 can also affect the MEK/ERK signaling pathway and influence tumor progression (Xue et al., 2019). The above findings suggest that HAVCR1 has huge prospects in tumor research as a potential target for antitumor therapeutics.

Herein, we analyzed TCGA data and found that HAVCR1 was overexpressed in tumor tissues of Pancreatic adenocarcinoma, Liver hepatocellular carcinoma, Esophageal carcinoma and Stomach adenocarcinoma compared to normal tissue, suggesting HAVCR1 is an important risk factor in these tumors and could serve as a prognostic biomarker. No changes in HAVCR1 expression were observed in different tumor stages, implying that HAVCR1 expression is not related to tumor staging.

Current evidence suggests that DNA methylation is negatively correlated with gene expression (Smith et al., 2020). Moreover, it has been shown that promoter hypermethylation is negatively correlated with gene expression, internal methylation is weakly negatively correlated with gene expression, and promoter hypomethylation is positively correlated with transcriptional activity (Hogg et al., 2020; Zhao et al., 2021). Accordingly, DNA methylation is an important mechanism for regulating gene expression, especially in genetic diseases and tumorigenesis (Vukic and Daxinger, 2019; Martisova et al., 2021). In the present study, we found that the promoter methylation of HAVCR1 was decreased in Liver hepatocellular carcinoma and Pancreatic adenocarcinoma.

Interestingly, the HAVCR1 ectodomain can affect the activation and function of immune cells (Telford et al., 2017). Infiltrated immune cells play an important role in the antitumor immune response; the numbers and types of tumor-infiltrating immune cells can determine the outcome of tumor immunity to a certain extent (Tan et al., 2021; Wang et al., 2021). It is currently unclear whether HAVCR1 expression can affect tumor infiltration of immune cells. Notably, HAVCR1 was associated with immune cell infiltration in Liver hepatocellular carcinoma and Pancreatic adenocarcinoma. Intriguingly, HAVCR1 expression significantly changed the tumor microenvironment, suggesting that HAVCR1 overexpression in tumors is associated with the invasion of non-tumor cells, such as immune cells and mesenchymal cells. There is overwhelming evidence that these non-tumor cells play an important role in tumorigenesis, development and drug resistance (Burgos-Panadero et al., 2019; Eiro et al., 2019; Jayant et al., 2020). In this regard, the extracellular matrix or mesenchymal cells have been documented to promote tumor proliferation and metastasis (Holmström et al., 2020; Cox, 2021). The non-tumor cells in the tumor microenvironment may provide new insights into tumor biology to develop predictive and prognostic models.

Many costimulatory molecules have been shown to exhibit synergistic or antagonistic effects with HAVCR1. In our study, we found that HHLA2, CD44 and TNFRSF4 were positively correlated with HAVCR1. HHLA2 is a newly discovered member of the B7 family (Zhu and Dong, 2018) that can inhibit the proliferation of CD4 and CD8 T cells and inhibit cytokine secretion by T cells (Zhao et al., 2013; Rieder et al., 2021). Mounting evidence suggests that the expression of HHLA2 in normal tissues is limited, and it is only expressed in epithelial cells. However, HHLA2 is highly expressed in various malignant tumors, which makes HHLA2 a potential therapeutic target for human tumors (Cheng et al., 2017; Chen et al., 2019; Jing et al., 2019). Importantly, HAVCR1 and HHLA2 have been suggested to exhibit a synergistic effect in tumorigenesis and development.

CD44 is a surface glycoprotein overexpressed in tumor cells. Hyaluronic acid (HA) is a major ligand for CD44, which can bind with other molecules, including metalloproteinases (MMPs), osteopontin and collagens (Senbanjo and Chellaiah, 2017). CD44 is expressed on tumor cell surfaces and combines with ligands, activating corresponding signals and participating in tumor cell proliferation, motility, survival, chemical resistance, and invasion (Hassn Mesrati et al., 2021). CD44 inhibition has been shown to enhance the chemosensitivity of tumor cells (Yoon et al., 2014).

Tumor necrosis factor receptor superfamily member 4 (TNFRSF4), also named OX40, is reportedly the immune checkpoint of the immune costimulatory pathway (Croft, 2010; Willoughby et al., 2017; Buchan et al., 2018). TNFRSF4 is mainly expressed in activated CD4^+^ and CD8^+^ T cells (Lu, 2021). TNFRSF4 combined with ligands can promote the clonal proliferation of T cells, enhance T cell memory, proliferation, immune surveillance and prevent the formation of immune tolerance (So and Ishii, 2019). In addition, TNFRSF4-positive T cells can reduce inhibitory factors in the tumor immune microenvironment and effectively inhibit tumor invasion and metastasis (Xie et al., 2010).

Tumor immunotherapy is an innovative approach to enhance the immune function of patients and achieve the ultimate goal of killing tumor cells (Jin et al., 2022). We found the HAVCR1 overexpressed in tumor tissue and participated in the immune response, which could be a potential tumor immunotherapy target, such as PD1, PDL1, CTLA4, p53, etc (Chasov et al., 2021; Jin et al., 2022). In recent years, CAR-T cell therapy has developed rapidly in tumor treatment, with excellent results in treating blood tumors (Hou et al., 2021). HAVCR1 overexpressed on the surface of tumor cells could also be an excellent tumor antigen to use in CAR-T cell therapy.

## Conclusion

Overall, our findings suggest that HAVCR1 upregulation is associated with a high risk for Liver hepatocellular carcinoma and Pancreatic adenocarcinoma and correlates with poor survival. The interplay among HAVCR1, HHLA2, CD44 and TNFRSF4 may play an important role in tumorigenesis and development. Importantly, HAVCR1 has huge prospects as a potential target of immune checkpoint blockade therapy in Liver hepatocellular carcinoma and Pancreatic adenocarcinoma patients.

## Data Availability

Publicly available datasets were analyzed in this study. This data can be found here: https://www.cancer.gov/about-nci/organization/ccg/research/structural-genomics/tcga.
