# Corrigendum: A comprehensive analysis of HAVCR1 as a prognostic and diagnostic marker for pan-cancer

**DOI:** 10.3389/fgene.2024.1357409

**Published:** 2024-01-26

**Authors:** Sheng Liu, Wenting Tang, Jing Cao, Mei Shang, Sun Hengchang, Jiao Gong, Bo Hu

**Affiliations:** ^1^ Department of Laboratory Medicine, Third Affiliated Hospital of Sun Yat-sen University, Guangzhou, China; ^2^ State Key Laboratory of Oncology in South China, Collaborative Innovation Center for Cancer Medicine, Sun Yat-sen Cancer Center, Guanghzou, China; ^3^ Department of Molecular Diagnostics, Sun Yat-sen University Cancer Center (SYSUCC), Guangzhou, China; ^4^ Department of Infectious Diseases, Third Affiliated Hospital of Sun Yat-sen University, Guangzhou, China

**Keywords:** HAVCR1, prognosis, diagnostic, liver hepatocellular carcinoma, pancreatic adenocarcinoma

In the published article, there was an error in the **Funding** statement. The funding number for The Science and Technology Program of Guangzhou was incorrectly given as “2019-02-01-06-3001-0005”. The correct number is “201903010039”. The corrected Funding statement appears below.

