# Supplementary material for: A Comprehensive Analysis of HAVCR1 as a Prognostic and Diagnostic Marker for Pan-Cancer
Source: Front Genet. 2022 Jun 8;13:904114. doi: 10.3389/fgene.2022.904114 (PMC9213751; doi:10.3389/fgene.2022.904114)
Supplement: Supplementary file 1 [file Table1.DOCX]

|  | **High (n=28)** | **Low (n=18)** | ***p*** |
| --- | --- | --- | --- |
| **Sex (Male, %)** | 26 (92.9) | 17 (94.4) | 1 |
| **Age (years)** | 57.54 (12.51) (12.51) | 55.78 (10.04) (10.04) | 0.619 |
| **HAVCR1 expression** | 2.39 (0.50) | 0.72 (0.46) | <0.001 |
| **Time (months)** | 24.68 (6.80) | 26.06 (6.60) | 0.501 |
| **Dead (%)** | 18 (64.3) | 7 (38.9) | 0.166 |
| **Antiviral treatment (%)** |  |  | 0.48 |
| **No** | 22 (78.6) | 13 (72.2) |  |
| **Yes** | 4 (14.3) | 3 (16.7) |  |
| **Iregular intake of antiviral drugs** | 1 (3.6) | 0 (0.0) |  |
| **Non-conventional antiviral therapy** | 0 (0.0) | 1 (5.6) |  |
| **Antiviral treatment with TCM Medicine** | 0 (0.0) | 1 (5.6) |  |
| **Treatment with self-made TCM formulas** | 1 (3.6) | 0 (0.0) |  |
| **Cirrhosis (Yes, %)** | 16 (57.1) | 8 (44.4) | 0.59 |
| **TNM stage ( II, %)** | 13 (46.4) | 9 (50.0) | 1 |
| **Recurrence (Yes, %)** | 7 (25.0) | 1 (5.6) | 0.194 |

Table 1. The correlation between clinical characteristics and HAVCR1 expression
